# Osteoblast-secreted collagen upregulates paracrine Sonic hedgehog signaling by prostate cancer cells and enhances osteoblast differentiation

**DOI:** 10.1186/1476-4598-11-30

**Published:** 2012-07-13

**Authors:** Samantha M Zunich, Maria Valdovinos, Taneka Douglas, David Walterhouse, Philip Iannaccone, Marilyn LG Lamm

**Affiliations:** 1 Department of Pediatrics, Northwestern University Feinberg School of Medicine, and Developmental Biology Program, Children’s Memorial Research Center, 2300 Children’s Plaza #204, Chicago, IL, 60614, USA

**Keywords:** Prostate cancer, Bone metastasis, Hedgehog, Collagen, Extracellular matrix, Osteoblast differentiation

## Abstract

**Background:**

Induction of osteoblast differentiation by paracrine Sonic hedgehog (Shh) signaling may be a mechanism through which Shh-expressing prostate cancer cells initiate changes in the bone microenvironment and promote metastases. A hallmark of osteoblast differentiation is the formation of matrix whose predominant protein is type 1 collagen. We investigated the formation of a collagen matrix by osteoblasts cultured with prostate cancer cells, and its effects on interactions between prostate cancer cells and osteoblasts.

**Results:**

In the presence of exogenous ascorbic acid (AA), a co-factor in collagen synthesis, mouse MC3T3 pre-osteoblasts in mixed cultures with human LNCaP prostate cancer cells or LNCaP cells modified to overexpress Shh (LNShh cells) formed collagen matrix with distinct fibril ultrastructural characteristics. AA increased the activity of alkaline phosphatase and the expression of the alkaline phosphatase gene *Akp2*, markers of osteoblast differentiation, in MC3T3 pre-osteoblasts cultured with LNCaP or LNShh cells. However, the AA-stimulated increase in *Akp2* expression in MC3T3 pre-osteoblasts cultured with LNShh cells far exceeded the levels observed in MC3T3 cells cultured with either LNCaP cells with AA or LNShh cells without AA. Therefore, AA and Shh exert a synergistic effect on osteoblast differentiation. We determined whether the effect of AA on LNShh cell-induced osteoblast differentiation was mediated by Shh signaling. AA increased the expression of *Gli1* and *Ptc1*, target genes of the Shh pathway, in MC3T3 pre-osteoblasts cultured with LNShh cells to at least twice their levels without AA. The ability of AA to upregulate Shh signaling and enhance alkaline phosphatase activity was blocked in MC3T3 cells that expressed a dominant negative form of the transcription factor GLI1. The AA-stimulated increase in Shh signaling and Shh-induced osteoblast differentiation was also inhibited by the specific collagen synthesis inhibitor 3,4-dehydro-L-proline.

**Conclusions:**

Matrix collagen, formed by osteoblasts in the presence of AA, potentiates Shh signaling between Shh-expressing prostate cancer cells and osteoblasts. Collagen and Shh signaling exert a synergistic effect on osteoblast differentiation, a defining event in prostate carcinoma bone metastasis. Investigations into paracrine interactions among prostate cancer cells, osteoblasts, and osteoblast-synthesized matrix proteins advance our understanding of mechanisms contributing to prostate cancer bone metastasis.

## Background

Prostate cancer is the second leading cause of cancer death among men in the United States, and there is clinical evidence of bone metastases in approximately 80 % of those who have died [1,2]. A comprehensive understanding of signaling interactions between invading epithelial-derived prostate cancer cells and the host bone stromal environment that promote bone metastasis is crucial to the development of effective therapeutic strategies.

Although markers of bone production and resorption may be present in patients, prostate carcinoma bone metastases are generally characterized by new bone formation initiated by the differentiation of mesenchymal progenitor cells into osteoblasts [3-5]. We have previously demonstrated that human prostate cancer cells, which express high levels of Sonic hedgehog (Shh), activate the signaling pathway in MC3T3 pre-osteoblasts and induce osteoblast differentiation [6].

Shh is a secreted glycopeptide that plays critical functions in the normal development of many organs including the prostate; and, deregulation of the Shh pathway has been linked to human cancer [7-9]. Expression of Shh and other members of the signaling pathway have been reported in human primary prostate carcinomas and metastases, including bone [10-13]. Paracrine induction of osteoblast differentiation via the Shh pathway may be a mechanism through which Shh-expressing prostate cancer cells initiate changes in the bone microenvironment that favor the development of metastases.

A hallmark of osteoblast differentiation both in vivo and in vitro is the formation of an extracellular matrix [14,15]. Type 1 collagen accounts for about 95 % of the organic matrix proteins in bone [4]. The role of matrix collagen in the formation of bone metastasis is not well understood.

In the present study, we investigated the formation of a collagenous ECM by osteoblasts induced to differentiate by Shh-expressing prostate cancer cells, and the effects of a collagenous matrix on paracrine signaling between prostate cancer cells and osteoblasts.

## Results

### Ascorbic acid promotes matrix collagen deposition in mixed cultures of osteoblasts and prostate cancer cells

We have previously used mixed cultures of mouse calvaria-derived MC3T3 pre-osteoblasts and human prostate cancer cells LNCaP genetically modified to overexpress Shh (designated LNShh cells) to demonstrate LNShh cell-mediated induction of osteoblast differentiation [6]. In the present studies, we utilized this in vitro mixed cell culture system to investigate the effects of an osteoblast-deposited collagen matrix on paracrine interactions between prostate cancer cells and differentiating osteoblasts. Mixed cultures were maintained for 7, 14, and 21 days to simulate temporal stages in the formation of a collagen matrix during osteoblast differentiation in vitro which mimics the in vivo process [14,16].

Ascorbic acid (AA) stimulates collagen matrix formation in primary cultures of fetal calvarial osteoblasts and non-transformed osteoblast cell lines including MC3T3 cells [16-18]. AA is a required co-factor for the hydroxylation of procollagen chains, a necessary step for triple helix formation, secretion, and collagen fibril assembly [15,19].

**Figure 1:**
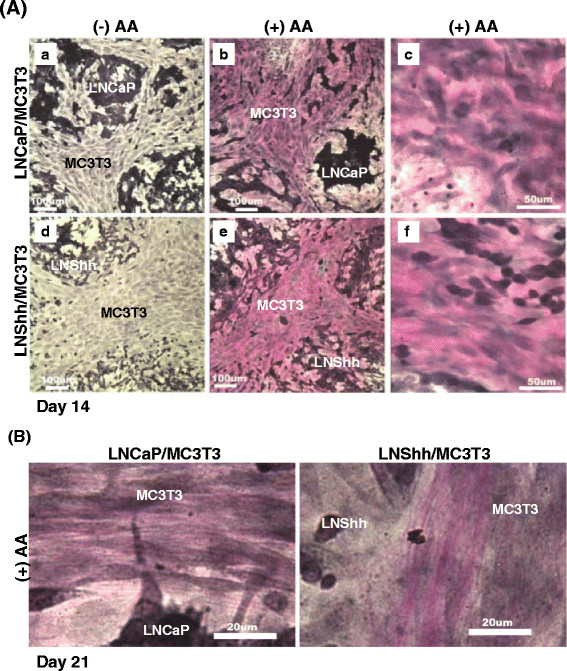
**AA promotes collagen deposition in mixed cultures of MC3T3 pre-osteoblasts and human prostate cancer cells.** In this and other figures, mouse MC3T3 pre-osteoblasts were mixed with either human LNCaP prostate cancer cells genetically modified to overexpress Shh (designated LNShh) or vector-transfected LNCaP cells as controls (designated LNCaP). Mixed cells were then seeded onto chamber slides, and cultures were maintained in AA-free α-MEM complete culture medium with or without exogenous AA [50 μg/ml in this and subsequent figures]. Mixed cultures were maintained for either 14 days (**A**) or 21 days (**B**) then stained with von Gieson stain for collagen. Bars = 100 μm in A(a, b, d, e); 50 μm in A(c and f); 20 μm in B.

In the absence of exogenous AA, von Gieson staining for collagen was insignificant in mixed cultures of MC3T3 pre-osteoblasts with either vector-transfected LNCaP cells (as controls) or LNShh cells through 14 days of culture (Figure 1A: *a* and *d*, respectively). Treatment with AA promoted collagen deposition in both mixed cultures (Figure 1A: *b* and *e*; *c* and *f*).

A difference in the appearance of matrix collagen was detected upon longer AA treatment. In contrast to the generally homogeneous staining of collagen in mixed cultures of MC3T3 and LNCaP cells treated with AA for 21 days, distinct strands of intensely stained collagen fibers were commonly observed in mixed cultures of MC3T3 and LNShh cells (Figure 1B).

### Differences in collagen fibril organization and diameter size distribution

**Figure 2:**
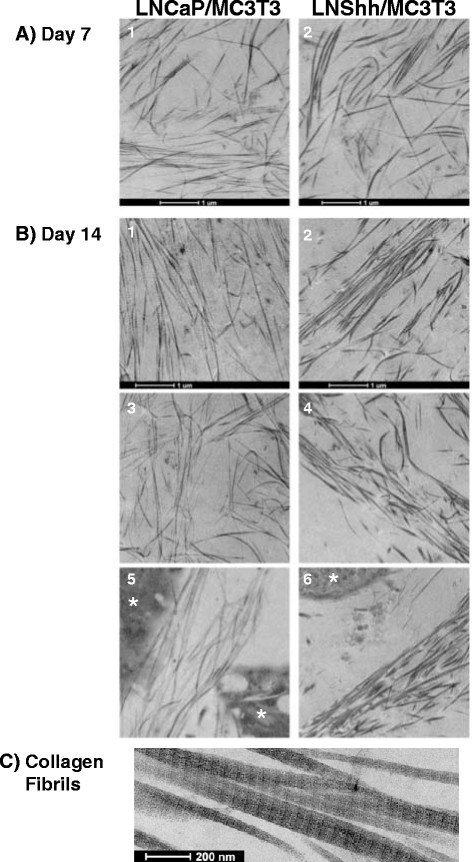
**Organization of matrix collagen fibrils in mixed cultures of MC3T3 pre-osteoblasts and human prostate cancer cells.** MC3T3 pre-osteoblasts were mixed with either LNCaP or LNShh cells then seeded onto Thermanox cover slips kept in 24-well plates. Mixed cultures were maintained for either 7 days (**A**) or 14 days (**B**) with exogenous AA. Mixed cultures were fixed and processed for TEM, and digital micrographs were captured as described under Materials and Methods. Results are representative of 2–9 TEM images from different grid sections of 1–3 samples per group. Bars = 1 μm (**A, B**) and 200 nm (**C**). *denotes cells.

To better characterize their ultrastructural properties, collagen fibrils in AA-treated mixed cultures were analyzed by transmission electron microscopy (TEM). At 7 days, collagen fibrils appeared randomly oriented in the matrix of mixed cultures of MC3T3 with either LNCaP or LNShh cells (Figure 2A: *1* and *2*, respectively). By day 14, collagen fibrils in mixed cultures of MC3T3 and LNCaP cells remained largely dispersed throughout the matrix with some fibrils in loose parallel alignment to one another (Figure 2B: *135*). In contrast, parallel-oriented collagen fibrils were prevalent in mixed cultures of MC3T3 and LNShh cells and appeared organized in distinct bundles (Figure 2B: *246*). Matrix fibrils in both mixed cultures displayed the banded pattern characteristic of native collagen (Figure 2C) [20].

**Figure 3:**
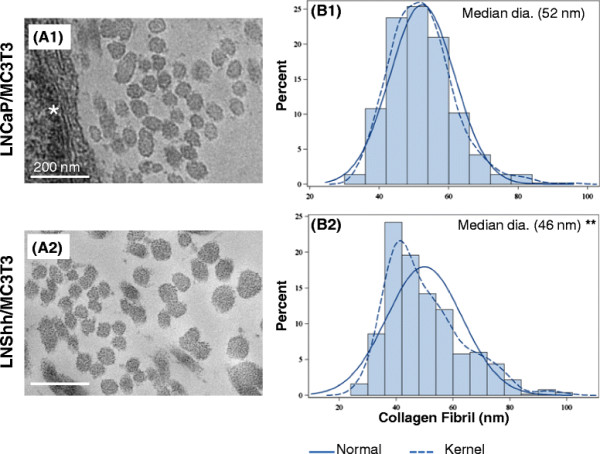
**Size distribution of collagen fibril diameters in mixed cultures of MC3T3 pre-osteoblasts and human prostate cancer cells.** MC3T3 pre-osteoblasts were mixed with either LNCaP or LNShh cells then seeded onto Thermanox cover slips and maintained for 21 days with exogenous AA. Mixed cultures were fixed and processed for TEM, and digital images were captured as described under Materials and Methods. (**A1, A2**) Results are representative of at least 7 TEM images from different grid sections of 3 samples per group. Bars = 200 nm. *denotes cells. (**B1, B2**) Density plots represent frequency distribution of fibril diameter sizes in mixed cultures. Normal and kernel density curves are superimposed. Data in each density plot were obtained from 500 fibrils randomly selected from 8–14 TEM images from different grid sections of 2 samples per group. **denotes significant difference in median fibril diameter size, *P < 0.001.*

Collagen fibril diameter size distribution was also different between mixed cultures as measured on day 21. As revealed in TEM images, matrix collagen fibrils in cultures of MC3T3 and LNCaP cells appeared more homogeneous in diameter size compared to those in cultures of MC3T3 and LNShh cells (Figure 3A1 and A2, respectively). Fibril diameter sizes of collagen fibrils in mixed cultures were measured and their frequency distributions are presented as density plots (Figure 3B1 and B2). Kernel density curves, obtained by kernel density estimation which is a non-parametric method of estimating the probability distribution of a random variable, were generated for each plot and compared to a normal distribution curve (Figure 3B1 and B2) [21]. Based on the asymptotic Kolmogorov-Smirnov two-sample test, the diameter size distributions of collagen fibrils in the two mixed cultures were statistically highly different (*P < 0.*0001). The unimodal distribution of fibril diameter sizes among collagen fibrils in mixed cultures of MC3T3 and LNCaP cells was almost identical to a normal distribution; whereas, the distribution in mixed cultures of MC3T3 and LNShh cells shifted toward the smaller fibril diameter range (Figure 3B1 and B2, respectively). The median diameter size of collagen fibrils in mixed cultures of MC3T3 and LNCaP cells was 52 nm (n = 500 fibrils) which is within range of the reported average diameter of collagen fibrils produced in vitro by MC3T3 cells (Figure 3B1) [22,23]. In contrast, the median diameter size of collagen fibrils in mixed cultures of MC3T3 and LNShh cells was significantly smaller at 46 nm (n = 500 fibrils; *P ≤ 0.001;* Figure 3B2).

Based on these data, we suggest that LNShh cells influence the morphological properties of collagen fibrils in matrix formed by differentiating osteoblasts.

### AA enhances LNShh cell-induced osteoblast differentiation

**Figure 4:**
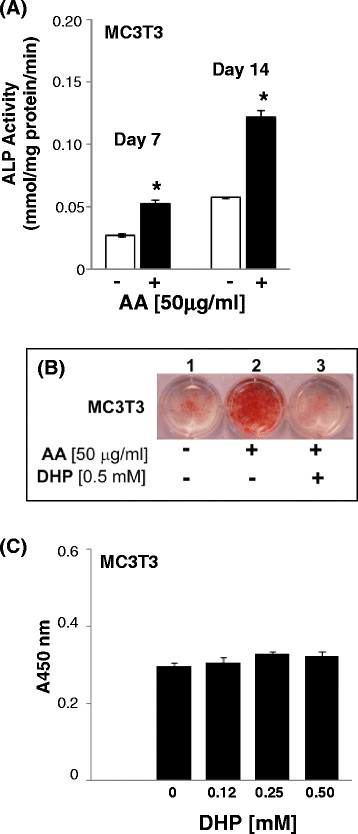
**AA increases ALP activity in MC3T3 pre-osteoblasts.** (**A**) MC3T3 cells were grown in 6-well plates for 7 or 14 days with or without exogenous AA. ALP activity was determined in cell lysates at indicated time points. ALP activity in the presence of AA (filled bars) was compared to that without AA (open bars) within each time point. Data are means ± SD of 4 replicates from 2 independent samples per group. *, *P < 0.05*. (**B**) MC3T3 cells were grown in 6-well plates for 14 days with or without AA and the collagen synthesis inhibitor 3,4-dehydro-L-proline (DHP; 0.5 mM). Cultures were then stained for ALP activity. Results are representative of 2 independent experiments. (**C**) MC3T3 cells were grown in 96-well plates for 24 h in the presence of varying concentrations of DHP. Absorbance measurements are in direct proportion to the number of living cells. Data are means ± SD of 4 replicate wells per treatment.

A well recognized outcome of the addition of AA to osteoblast cultures is the induction of osteoblast differentiation [16-18]. We confirmed this effect and showed that exogenous AA significantly increased alkaline phosphatase (ALP) activity, a hallmark of osteoblast differentiation, in cultures of MC3T3 pre-osteoblasts (Figure 4A).

AA promotes osteoblast differentiation of MC3T3 cells by enhancing collagen matrix accumulation [16,18]. Indeed, the AA-stimulated increased staining for ALP activity in MC3T3 pre-osteoblasts was dramatically inhibited by the specific collagen synthesis inhibitor 3,4-dehydro-L-proline (DHP) without significantly affecting cell proliferation (Figure 4B and C, respectively).

We have previously demonstrated that LNShh cells induce differentiation of MC3T3 pre-osteoblasts in the absence of exogenous AA via direct and specific paracrine activation of the Shh pathway [6]. Here, we investigated the combined effect of paracrine Shh signaling and AA on osteoblast differentiation.

**Figure 5:**
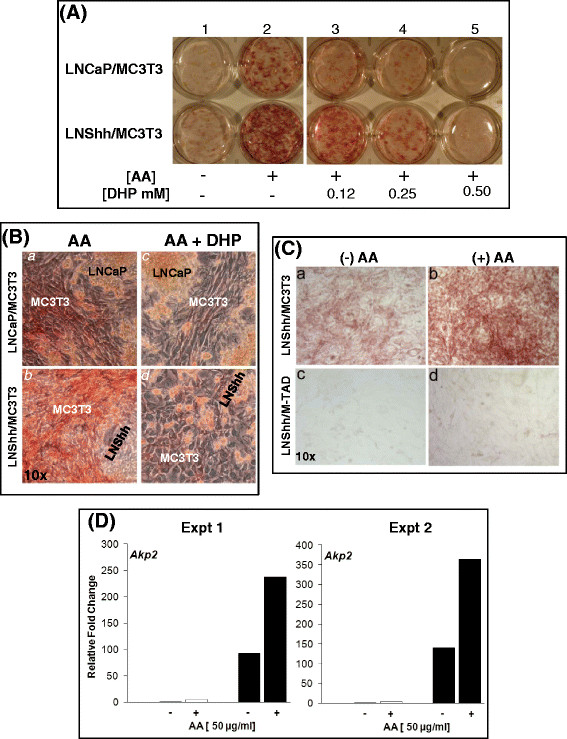
**AA enhances LNShh cell-induced osteoblast differentiation.** (**A, B**) MC3T3 cells were mixed with either LNCaP or LNShh cells then seeded onto 6-well plates. Mixed cultures were maintained for 14 days with or without exogenous AA and varying concentrations of DHP. Following, cultures were stained for ALP activity. In (**B**), cultures treated with AA in the presence or absence of 0.5 mM DHP were further examined under light microscopy to demonstrate staining for ALP activity in MC3T3 pre-osteoblasts. Magnification, 10x. Results are representative of 2 independent experiments. (**C**) LNShh cells were cultured with either MC3T3 cells that express the message for the human *GLI1(−TAD)* transgene (designated M-TAD cells) or parental MC3T3 cells as controls. Both MC3T3 and M-TAD cells express endogenous mouse *Gli1* message; but, only the M-TAD cells express the dominant negative form of the transcription factor *GLI1*[6]. Mixed cultures were maintained for 7 days with or without AA and stained for ALP activity. Magnification, 10x. Results are representative of 2 independent experiments. (**D**) Mixed cultures were maintained for 7 days with or without exogenous AA. In this and subsequent figures, gene expression in MC3T3 cells in mixed cultures was determined by quantitative real-time RT-PCR analysis using mouse species-specific gene primers [6]. Expression of *Akp2* in MC3T3 cells cultured with LNShh cells (filled bars) relative to those cultured with control LNCaP cells (open bars) were compared. Data from 2 independent experiments with similar results are presented.

In the absence of exogenous AA, staining for ALP activity was more evident in mixed cultures of MC3T3 and LNShh cells compared to that in cultures of MC3T3 and LNCaP cells (Figure 5A, lane 1). The addition of AA dramatically increased the staining for ALP activity in both mixed cultures (Figure 5A, lane *2*). However, staining in AA-treated cultures of MC3T3 and LNShh cells was more intense than that in AA-treated cultures of MC3T3 and LNCaP cells although confluent populations of MC3T3 pre-osteoblasts, in which staining was localized, were present in both (Figure 5B, *a-b*).

The AA-stimulated increased staining for ALP activity in both mixed cultures was blocked by DHP (Figure 5A: lanes *3*–*5*). The marked loss of AA-stimulated staining for ALP activity in both mixed cultures treated with 0.5 mM DHP was not due to loss of MC3T3 pre-osteoblasts since confluent populations of these cells were present in both (Figure 5B, *c-d*).

Significantly, the AA-stimulated increase in staining for ALP activity was inhibited in MC3T3 cells stably transfected with a dominant negative form of the Shh pathway transcription factor GLI1, designated M-TAD cells (Figure 5C, *b* and *d*).

The alkaline phosphatase gene *Akp2* is a marker gene of osteoblast differentiation. In MC3T3 cells cultured with LNCaP cells, *Akp2* expression was increased approximately 5-fold with AA treatment (Figure 5D: Expt 1 and Expt 2). Even without AA, *Akp2* expression in MC3T3 cells cultured with LNShh cells was approximately 100-fold greater than that in MC3T3 cells cultured with LNCaP cells, an indication of the effect of Shh signaling alone. AA further upregulated the *Akp2* expression in MC3T3 cells cultured with LNShh cells to greater than 200-fold, which far exceeded the levels observed in MC3T3 cells cultured with either LNShh cells without AA (Shh effect) or with LNCaP cells with AA (AA effect). We suggest based on these results that Shh and AA exert a synergistic effect on osteoblast differentiation.

### AA upregulates paracrine Shh signaling in MC3T3 pre-osteoblasts

We determined whether the synergistic effect of AA on LNShh cell-stimulated osteoblast differentiation was mediated through increased paracrine Shh signaling between prostate cancer cells and osteoblasts. To demonstrate paracrine activation of the pathway in mouse MC3T3 cells cultured with human LNCaP or LNShh cells, the expression of known Shh target genes *Gli1* and *Ptc1* in MC3T3 cells was determined by quantitative real-time RT-PCR analysis using species-specific primer sequences which amplified these genes in mouse cells but not in human prostate cancer cells [6,10]. Thus, amplification of *Gli1* and *Ptc1* by mouse species-specific primer sequences in mixed cultures is highly, if not solely, attributable to gene expression in MC3T3 pre-osteoblasts [6].

**Figure 6:**
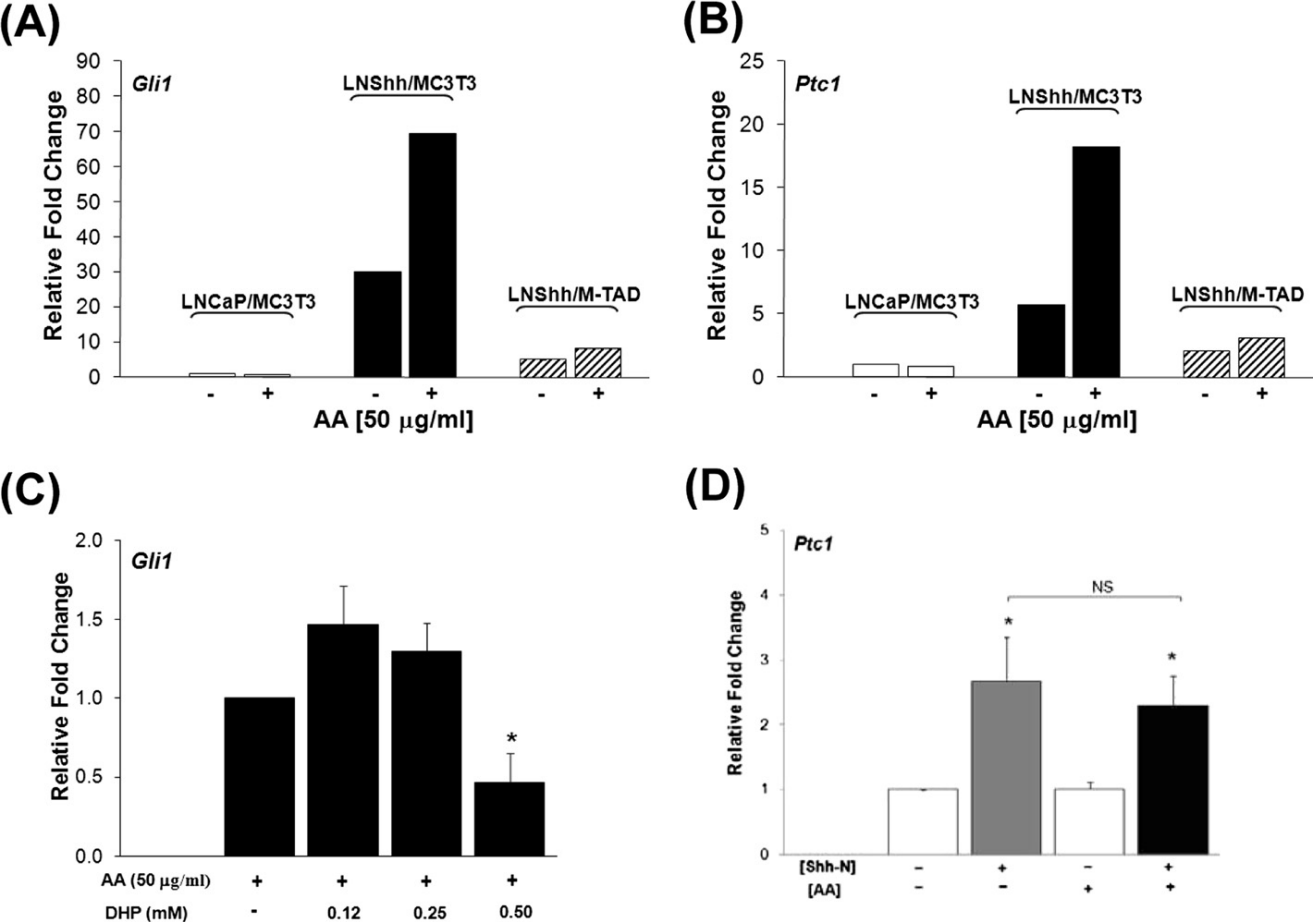
**AA upregulates paracrine Shh signaling between MC3T3 pre-osteoblasts and LNShh cells.** (**A** and **B**) Pre-osteoblasts [MC3T3 and M-TAD cells] were mixed with either LNCaP or LNShh cells as indicated and maintained for 7 days with or without exogenous AA. Results are representative of 2–4 assays from 2 independent experiments, each assay done at least in duplicates. (**C**) Expression of *Gli1* in MC3T3 cells cultured with LNShh cells for 14 days in the presence of AA and varying concentrations of DHP were compared. Data are means ± SD of 3 assays from 2 independent experiments, each assay done at least in duplicates. (**D**) MC3T3 cells were cultured alone for 24 h with or without 1 μg/ml Shh-N and 50 μg/ml AA. *Ptc1* expression levels among groups were compared. All values are means ± SD from 2 independent experiments. *, *P < 0.05.*

In the absence of exogenous AA, the expression levels of *Gli1* and *Ptc1* were markedly upregulated in MC3T3 cells cultured with LNShh cells compared to those cultured with LNCaP cells (Figure 6A and B, respectively). These results are consistent with our previous findings [6].

AA did not increase the basal expression of *Gli1* and *Ptc1* in MC3T3 pre-osteoblasts cultured with control LNCaP cells. Interestingly, AA further increased the *Gli1* and *Ptc1* levels by at least 2-fold in MC3T3 cells cultured with LNShh cells (Figure 6A and B, respectively). The AA-stimulated increases in *Gli1* and *Ptc1* expression levels were blocked in M-TAD cells which express a dominant negative form of the transcription factor GLI1.

To determine whether the AA-promoted increase in paracrine activation of the Shh pathway was mediated through AA’s actions on collagen synthesis and matrix deposition, mixed cultures of MC3T3 and LNShh cells were treated with exogenous AA with or without DHP. As shown in Figure 6C, DHP abrogated the AA-stimulated increase in *Gli1* expression in MC3T3 pre-osteoblasts cultured with LNShh cells. Similar effects were observed with *Ptc1* expression (data not shown).

To determine whether AA might also directly influence the Shh pathway, single cultures of MC3T3 pre-osteoblasts were treated with AA in the absence or presence of exogenous Shh peptide. *Ptc1* expression was significantly upregulated following treatment with Shh peptide alone but not with AA alone (Figure 6D). The Shh peptide-stimulated increase in *Ptc1* message was not increased further by combined treatment with AA. Similar results in *Gli1* expression were obtained (data not shown).

## Discussion

We provide novel evidence that AA potentiates Shh signaling between prostate cancer cells and osteoblasts, and synergistically enhances Shh-induced osteoblast differentiation.

The ability of AA to upregulate Shh signaling in osteoblasts requires Gli transcriptional activity. The expression of target genes *Gli1* and *Ptc1* in MC3T3 pre-osteoblasts cultured with LNShh cells was increased by exogenous AA to at least twice their levels in the absence of AA. However, the effect of AA was blocked in MC3T3 pre-osteoblasts that expressed a dominant negative form of GLI1, the M-TAD cells. The GLI1 translated product in M-TAD cells is expected to bind to the consensus DNA GLI binding site but not activate the pathway [6]. AA does not directly impact Gli transcriptional activity since treatment with AA did not increase the expression of *Ptc1 (*and *Gli1*; not shown) in MC3T3 pre-osteoblasts cultured alone; whereas, exogenous Shh peptide did. And, the combined treatment of AA and Shh peptide did not significantly upregulate gene expression above that attained with exposure to the Shh peptide alone. In agreement with our earlier observations, we indicate an indirect mechanism of AA action on the Shh pathway [6].

The effect of AA on Shh signaling requires collagen production. The specific collagen synthesis inhibitor DHP blocked the AA-promoted upregulation of *Gli1* in MC3T3 pre-osteoblasts cultured with LNShh cells. DHP is a proline analog which incorporates into nascent pro-α collagen chains and prevents prolyl hydroxylation, a critical step in procollagen triple helix formation and secretion [24]. The AA-stimulated incorporation of ^3^ H]proline into α_1_(I) and α_2_(I)-procollagen molecules in MC3T3 cells was blocked by DHP at 0.5 mM [16], the same concentration that inhibited AA-mediated upregulation of the Shh pathway in MC3T3 cells in the present studies.

The mechanism for collagen-mediated augmentation of Shh signaling is not clear. Collagen actions are mediated via the integrin signaling pathway. Integrins are cell-surface heterodimers consisting of α and β subunits. Type 1 collagen binds most commonly to cell surface integrins: α_1_β_1_ and α_2_β_1_[25]. The interaction between type 1 collagen and osteoblast integrins activate downstream cascades including the mitogen-activated protein kinase and phosphatidylinositol 3-kinase pathways, which lead to increased expression of target genes [26,27]. Recently, these pathways have been implicated in non-canonical activation of Shh signaling [28,29]. It is intriguing to suppose that intracellular cross-talk between the downstream mediator(s) of collagen-integrin signaling and the Shh pathway may have contributed to increased transcriptional activation of *Ptc1* and *Gli1* message in differentiating osteoblasts.

However, a role for collagen in the upregulation of the Shh pathway in osteoblasts must involve, at least in part, a Shh ligand-activated mechanism i.e., canonical hedgehog signaling. The upregulatory effect of AA on the Shh pathway was observed in MC3T3 pre-osteoblasts cultured with LNShh cells but not with LNCaP cells although matrix collagen was present in both mixed cultures. The collagen matrix may sequester Shh ligands and present them more effectively to Ptc receptors on target cells. Investigators have shown that the activity of ligands stored in the ECM such as transforming growth factor-β1 (TGF-β1) and bone morphogenetic proteins (BMPs) is influenced by matrix proteins including collagen [30-32]. Additionally, there is evidence that Shh molecules bind to ECM components vitronectin and laminin [33,34]. Although binding of Shh to collagen has yet to be demonstrated, the orientation and/or proximity of Shh molecules to Ptc receptors on osteoblasts might be optimized when the ligands are complexed with collagen. Collagen might also enhance the expression and/or function of membrane-bound components of the pathway including the Ptc receptor and Smoothened (Smo) leading to increased activation of Shh signaling. The cell surface expression of ligand-binding TGF-β1 receptors in MC3T3 cells was downregulated by AA treatment; and, this effect was linked to AA’s action on the synthesis of collagen and its interaction with α_2_β_1_ integrins [30]. Further investigations are needed to unravel the mechanisms through which collagen activates the Shh pathway.

We have previously shown that paracrine activation of the Shh pathway induces osteoblast differentiation in the absence of exogenous AA [6]. We have confirmed these findings and further showed that AA-enhanced osteoblast differentiation requires Gli transcriptional activation since M-TAD cells failed to respond to the osteogenic effect of LNShh cells. More importantly, we have demonstrated a synergistic effect of AA on Shh-induced osteoblast differentiation. Levels of *Akp2* expression in MC3T3 pre-osteoblasts exposed to both Shh signaling and AA (i.e., cultured with LNShh cells and treated with AA) were markedly greater than those in MC3T3 cells exposed to Shh alone or AA alone. The effect of AA is linked to the presence of matrix collagen since the collagen synthesis inhibitor DHP blocked the AA-promoted upregulation of ALP activity in MC3T3 osteoblasts cultured with LNShh cells. These findings are consistent with previous reports of collagen-dependent synergistic interactions between AA and osteogenic stimuli including BMP2 and interleukin-11 [31,35,36].

The biological significance of collagen fibril structural properties in cancer development and progression is just beginning to be recognized. Distinct signatures of collagen fibril organization were observed in the stromal environment of breast tumor tissues, and collagen fiber alignment appeared to correlate with tumor cell invasiveness [37]. Cancer cells, particularly metastatic cells, move linearly along collagen fibers [38]. Conceivably, the parallel alignment of collagen fibrils in mixed cultures of MC3T3 pre-osteoblasts and LNShh cells could facilitate prostate cancer cell motility and migration. The higher percentage of collagen fibrils with smaller diameter sizes in mixed cultures of MC3T3 and LNShh cells might indicate a more dynamic process of fibril assembly and matrix organization where early stage small-diameter fibril intermediates are continuously formed [39,40]. Further studies to determine the role of Shh-expressing prostate cancer cells in regulating the structural properties of bone matrix collagen, particularly under in vivo conditions, will increase our understanding of the significance of the Shh pathway in shaping the bone stromal microenvironment to support metastasis.

The role of matrix collagen on cell functions has been investigated mostly through use of scaffolds formed by either commercially-available, laboratory-prepared native type 1 collagen extracted from rat tail tendons, or collagen formed by osteoblasts. In these studies, cells are either added onto the gellified matrix or mixed with the matrix as it solidifies. Thus, matrix density and collagen fibril structural characteristics are largely pre-determined for the inoculated cells. Our mixed cell culture system enables both cancer cells and osteoblasts to interact during the process of AA-dependent collagen matrix formation. This allows investigations into reciprocal in situ interactions among cancer cells, osteoblasts, and osteoblast-synthesized matrix proteins that should provide significant insights into signaling processes relevant to bone metastasis.

## Conclusions

AA-induced formation of collagen matrix potentiates paracrine Shh signaling between prostate cancer cells and osteoblasts, and synergistically enhances Shh-induced osteoblast differentiation, an early and defining event in prostate carcinoma bone metastasis. Co-targeting of the Shh pathway and processes that regulate collagen matrix formation in bone presents a viable therapeutic approach against bone metastasis.

## Methods

### Cells and plasmid transfections

Parental LNCaP human prostate cancer cells and mouse calvaria-derived non-transformed pre-osteoblast cells MC3T3-E1 (subclone 4; designated as MC3T3 cells) were commercially obtained (ATCC, Rockville, MD).

LNCaP cells have been previously stably transfected with a 1.44 kb human *Shh* cDNA cloned into a pIRES2-EGFP mammalian cell expression vector (designated LNShh cells) or with pIRES2-EGFP vector alone as controls (designated LNCaP cells) [10]. We have previously confirmed the increased expression of Shh at the gene and protein levels in LNShh cells compared to LNCaP cells [10]. The morphology of LNCaP and LNShh cells appears identical and these cells exhibit similar growth properties in culture [10,41]. LNCaP and LNShh cells were maintained at 37 C, 5 % CO_2_ in complete culture medium consisting of RPMI-1640 supplemented with 10 % fetal bovine serum (FBS), 100 U/ml penicillin and 100 μg/ml streptomycin (Gibco Invitrogen). Shh gene and protein expression were routinely determined by quantitative real time RT-PCR and western blot analysis, respectively, and GFP expression was monitored by fluorescence microscopy.

MC3T3 cells have been previously stably transfected with pCMV-*GLI*(−)TAD: a human *GLI1* cDNA lacking a transactivation domain and cloned into pcDNA3 plasmid (designated M-TAD cells) [6]. We have previously shown that both M-TAD cells and parental MC3T3 cells (used as controls) express endogenous mouse *Gli1* message; but, only the M-TAD cells express the message for the human *GLI1(−TAD)* transgene whose GLI1 translated product is expected to bind to the consensus DNA GLI binding site but not activate the pathway; thus, acting as a dominant negative transcription factor [6]. MC3T3 and M-TAD cells were maintained at 37 C, 5 % CO_2_ in non-differentiation complete culture medium consisting of ascorbic acid (AA)-free α-MEM supplemented with 10 % FBS, 100 U/ml penicillin and 100 μg/ml streptomycin (Gibco Invitrogen).

### Mixed culture of cells

LNCaP or LNShh cells (5 × 10^4^) and MC3T3 or M-TAD cells (0.5 × 10^4^) were mixed in AA-free α-MEM complete culture medium and seeded per well of 6-well tissue culture plates. When grown in chamber slides, pre-osteoblasts and prostate cancer cells were mixed at equal concentrations of 1 × 10^4^ cells per cell line. Cultures were maintained for the length of time specified in the experiments with media changes every 2–3 days.

### Effect of ascorbic acid

L-ascorbic acid (AA; Aldrich) was dissolved in AA-free α-MEM complete culture medium at 50 μg/ml final concentration. Mixed cultures were maintained in complete culture medium with AA or in complete culture medium only as controls. The AA concentration used in these experiments is below pharmacologic concentrations that may be cytotoxic to prostate cancer cells including LNCaP [42,43]. To determine the direct effect of AA, MC3T3 cells were seeded alone onto 6-well culture plates at 1 × 10^5^ cells per well and treated with AA as above.

### Effect of Shh peptide

Shh-N, a modified active N-terminal peptide of human Shh (kindly provided by Curis Inc., Cambridge, MA), was prepared in serum-free AA-free α-MEM culture medium at 1 μg/ml final concentration. MC3T3 cells were seeded onto 6-well tissue culture plates at 1 × 10^5^ cells per well in AA-free α-MEM complete culture medium. Following overnight incubation, cells were maintained for 24 h in serum-free culture medium with Shh-N or in serum-free culture medium only as controls.

### Effect of collagen synthesis inhibitor

The collagen synthesis inhibitor 3,4-dehydro-L-proline (DHP; Sigma) was dissolved in AA-free α-MEM complete culture medium. Mixed cultures were maintained in complete culture medium with 50 μg/ml AA and varying concentrations of DHP or in complete culture medium only (i.e., without both AA and DHP) as controls. In some experiments, single cultures of MC3T3 cells were used.

### RNA isolation and real time quantitative RT-PCR

Total RNA was extracted using Trizol (Invitrogen), purified using the RNeasy Mini Kit (Qiagen) and subjected to DNase treatment with RQ1 RNase-free DNase (Promega) to remove contaminating genomic DNA. The TaqMan® Gold PCR Core Reagent Kit along with MuLV Reverse Transcriptase and RNase Inhibitor (Applied Biosystems) were used for cDNA synthesis. PCR primers (Invitrogen) and FAM-QSY7 probes (MegaBases, Inc.) for genes of interest and the housekeeping gene glyceraldehyde-3-phosphate dehydrogenase were designed using the Primer Express 3.0 software program. Mouse species-specific primer sequences, which amplified genes of interest in mouse MC3T3 but not in human prostate cancer cells, have been published [6]. mRNA expression was measured in duplicate or triplicate per sample using 40 cycles of amplification in the 7500 Fast Real-Time PCR System (Applied Biosystems). Reactions were routinely performed without Reverse Transcriptase to demonstrate RNA dependence of the reaction products. Results were analyzed using the comparative C_t_ method as described previously [6]. Data are expressed as relative fold change in gene expression.

### Cell proliferation assay

MC3T3 cells were seeded onto 24-well tissue culture plates at 0.2 × 10^4^ cells per well and maintained in AA-free α-MEM complete culture medium. Proliferation was determined using the Cell Counting Kit-8 (Dojindo Laboratories, Japan) which is based on the formation of a water-soluble formazan dye through the activity of dehydrogenases in living cells. Absorbance measurements at 450 nm are in direct proportion to the number of living cells.

### Immunocytochemistry

Cells were maintained as mixed cultures in Lab-TekII CC2-treated chamber slides (Nunc) for the length of time specified in the experiments with media changes every 2–3 days. Cells were fixed in 10 % neutral buffered formalin for 10 minutes and processed for von Gieson staining for collagen. For positive control, a section of aorta was similarly processed and stained. Slides were viewed in a Leica DMR-HC Upright Microscope and images were captured with imaging software (Improvision Openlab).

### Alkaline phosphatase activity

Quantitative determination of ALP activity was done using the p-Nitrophenyl Phosphate (pNPP) Liquid Substrate System (Sigma Aldrich) as previously described [6]. Absorbance at 405 nm was measured using a microplate reader, and ALP activity was calculated according to manufacturer’s instructions. Protein determination was done using the Bio-Rad *DC* Protein Microplate Assay according to manufacturer’s protocol.

Staining for ALP activity was performed on mixed cultures which were fixed with 10 % neutral buffered formalin for 10 minutes and incubated with alkaline phosphatase substrate solution (Sigma-Aldrich) for at least 30 minutes at room temperature in the dark as previously described [6].

### Transmission electron microscopy

Mixed cultures of MC3T3 pre-osteoblasts and LNCaP or LNShh cells were maintained in the presence of AA (50 μg/ml) on Thermanox cover slips (13 mm diameter; Electron Microscopy Services) kept in 24-well tissue culture plates for 7, 14, and 21 days. At end of culture, samples were fixed overnight at 4 C in 0.1 M sodium cacodylate buffer pH7.3 containing 2 % paraformaldehyde and 2.5 % glutaraldhyde, post-fixed with 2 % osmium tetroxide in 0.1 M sodium cacodylate buffer, and rinsed with distilled water. Following, samples were stained en bloc with 3 % uranyl acetate, rinsed in distilled water, dehydrated in ascending grades of ethanol, embedded in resin mixture of Embed 812 and Araldite, and cured in oven at 60 C. Samples were sectioned on a Leica Ultracut UC6 ultramicrotome, and 70 nm thin sections were collected on 200 mesh copper grids and post-stained with 3 % uranyl acetate and Reynolds lead citrate. Samples were sectioned either parallel to cell layers to reveal fibril orientation or perpendicular to cell layers to allow morphometric measurement of fibril diameters [23,39]. Samples were examined on FEI Tecnai Spirit G2 TEM, and digital images were captured on an FEI Eagle camera at magnifications ranging from 1900x to 49000x. Two to three grids per sample were examined and images from several sections per grid were taken.

### Analysis of collagen fibril diameter

Diameters of collagen fibrils were measured, using the analysis measurement tool of the Adobe Photoshop CS3 Extended software, on TEM images of samples sectioned perpendicular to cell layers and examined at magnification of 49000×. Fibril diameters were measured from 500 randomly selected collagen fibrils from 8–14 TEM images from different grid sections from each of 2 samples per group.

### Data analysis

Data were analyzed by ANOVA and pair-wise multiple comparisons were done using the Bonferroni *t*-test at *P < 0.05*. Comparison between two groups was done by Student’s *t*-test. Fibril diameter size distributions were compared using the asymptotic Kolmogorov-Smirnov two-sample test [21].

## Competing interests

The authors declare that they have no competing interests.

## Authors' contributions

SMZ performed experiments and helped with data analysis. MV and TD performed experiments and contributed to gene expression data analysis. DW and PI contributed to data analysis and manuscript editing. MLGL designed the studies, performed experiments, performed data analysis, and prepared the manuscript. All authors read and approved the final manuscript.
